# The Invasive Nearctic Pest *Platynota stultana* Walsingham (Lepidoptera: Tortricidae) Is Established in Southern Italy

**DOI:** 10.3390/insects17010122

**Published:** 2026-01-21

**Authors:** Lorenzo Goglia, Giorgio Formisano, Valentino Maria Guastaferro, Lorenza Albano, Domenico Giuseppe Crispo, Raffaele Griffo, Gennaro Di Prisco, Massimo Giorgini

**Affiliations:** 1 National Research Council of Italy, Institute for Sustainable Plant Protection, Piazzale Enrico Fermi, 1, 80055 Portici, Italy; giorgio.formisano@cnr.it (G.F.); valentinomariaguastaferro@cnr.it (V.M.G.); lorenzaalbano@cnr.it (L.A.); domenicogiuseppecrispo@cnr.it (D.G.C.); gennaro.diprisco@cnr.it (G.D.P.); 2 Plant Protection Service of Campania Region, via G. Porzio, Centro Direzionale Is. A6, 80143 Naples, Italy; lellogriffo@libero.it

**Keywords:** alien species, COI gene sequencing, DNA barcoding, invasion route, mitochondrial DNA, molecular phylogenetic analysis, omnivorous leafroller, quarantine pest

## Abstract

*Platynota stultana* is a Nearctic moth of economic importance for many crops in North America. It is a quarantine pest in Europe, where Mediterranean regions, with warm climates similar to those of the moth’s native range, are at risk of invasion. To date, the species is established only in Spain. It has been reported sporadically in Italy, but it is unknown whether these were transient findings or the result of an establishment. In this study, the presence of *P. stultana* in the Campania region, Southern Italy, was recorded. Adults of both sexes were found in different locations and in two consecutive years, suggesting that the species is established. Sequencing the COI gene identified three haplotypes of *P. stultana*, suggesting possible multiple introductions. The two most numerous haplotypes were identical to haplotypes from Florida. Phylogenetic analysis showed that the *P. stultana* clade splits into two subclades. The Italian haplotypes are all grouped into the same subclade. Our data suggest that *P. stultana* is expanding its range of invasion into Southern Italy, where, due to global warming, it may find increasingly favorable conditions and become an economic pest. A monitoring plan is required to allow timely implementation of control measures.

## Simple Summary

*Platynota stultana* is a Nearctic moth known to be a polyphagous pest of economic importance that targets many horticultural, fruit, and flower crops in North America. Its introduction into warm climate regions, through the trade of agricultural products, is feared. It is a quarantine pest worldwide. The Mediterranean Basin, with regions characterized by a climate close to that of its native range (South-Western USA and Mexico), is at risk of invasion. In this area, to date, the species is only established in Spain, where it is of limited economic interest. In Italy, *P. stultana* has been reported sporadically several times, but it is unknown whether these records are transient findings or the result of an establishment. In this study, we show that *P. stultana* has become established in Southern Italy. Through sequencing and phylogenetic analysis of the mitochondrial COI gene, we characterized the genetic diversity of the sampled populations and their geographical origins (predominantly Florida and California). Due to global warming, which is severely affecting the Mediterranean Basin, *P. stultana* could find better conditions under which to expand its range and become an economic pest. Our results indicate a need to implement an adequate monitoring plan in Southern Italy to allow timely planning of control measures.

## 1. Introduction

*Platynota stultana* Walsingham (Lepidoptera, Tortricidae, Sparganothini), known as the “omnivorous leafroller”, was originally described in Sonora (Mexico), but it is thought to also be native to Arizona [1]. In North America, where it is widely distributed, *P. stultana* is an economically important pest, especially in the Southern USA and Mexico [1]. It is a highly polyphagous species, having been reported on over 100 cultivated and wild plant species belonging to 30 different families, frequently Asteraceae and Fabaceae [2,3,4,5]. It is found on numerous fruit and vegetable crops, where the larvae feed on leaves and fruit. On the latter, the larvae generally cause surface erosion, resulting in a reduction in the fruit’s market value. Economic damage has been reported on cotton, peppers, grapes, citrus, stone fruits, pome fruits, and berries [6]. Damage may be particularly heavy in vineyards and berry crops because the larvae can also penetrate the fruit, promoting the development of fruit rot [6,7]. Finally, it has also been reported to be a pest that affects greenhouse rose and carnation cultivation [8]. Due to its wide plant-host range, the risk of introduction and redistribution of *P. stultana*, through the trade of agricultural products, is high. Indeed, it is formally considered a quarantine pest worldwide, including in European and South American countries, Morocco, China, and Japan [6,9,10]. In the EU, the European Plant Protection Organization (EPPO) includes *P. stultana* in the A2 list of pests recommended for regulation as quarantine pests [6].

The first report of *Platynota stultana* in Europe came from the United Kingdom, where a single larva was discovered in 2004 on a *Lantana* sp. plant imported from the United States [11]. Subsequently, it was reported infesting greenhouse pepper crops in Southern Spain [12]. In 2018, a pupa was intercepted in Germany on plants originating from Spain [13]. A few samples were trapped in 2022 in Western France [14]. Recently, its presence was confirmed in Malta [15], Portugal, and Greece [16]. However, to date, *P. stultana* appears to be established only in Southern Spain [6,17].

In Italy, following the first report of adult male *P. stultana* specimens in the region of Apulia, in the south [13,18], the moth has since been recorded multiple times across the peninsula and on the island of Sicily [16]. However, it remains unclear whether these records represent transient finds or indicate the establishment of the species. In this study, we documented the first record of *P. stultana* in the Campania region of Southern Italy, using multiple collection methods and combining morphological and molecular data for taxonomic identification of the specimens. Furthermore, we attempted to trace the species’ invasion route. Adults of both sexes were found at different locations and under various environmental conditions over two consecutive years, suggesting that the species is established in Southern Italy and is expanding its invasion range.

## 2. Materials and Methods

### 2.1. Insect Collection

**Figure 1:**
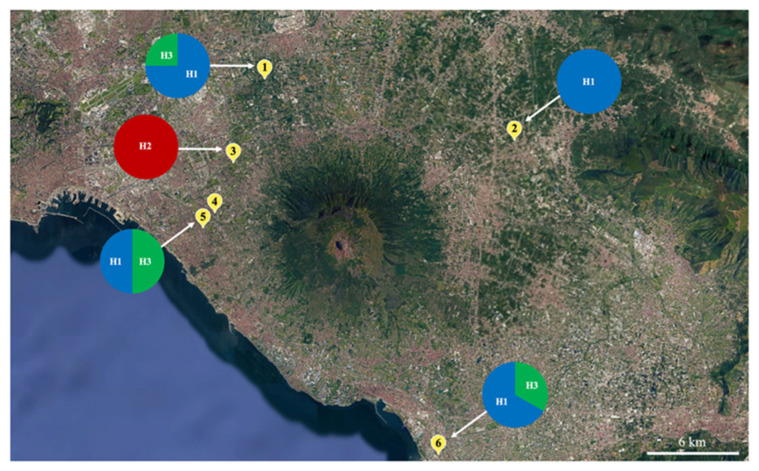
Collection sites in the Campania region of Southern Italy, along with the frequency with which *Platynota stultana* haplotypes occur. The sites are marked on the map with numbers from 1 to 6 (1, Santa Anastasia; 2, San Gennaro Vesuviano; 3, Cercola; 4, San Giorgio a Cremano; 5, Portici; and 6, Castellammare di Stabia). H1, H2, and H3 are the haplotypes of *P. stultana* identified based on the sequencing of the mitochondrial COI gene. The pie charts represent the frequency with which the haplotypes occur [Map data © 2025 Google].

The insects studied were sampled during faunistic surveys conducted in the province of Naples using three different collection methods, namely, UV light traps, sweeping, and rearing of wild plants. The geographic distribution of the collection sites is shown in Figure 1. A UV light trap, assembled according to the method reported by Infusino et al. [19], was placed in a field at night in October 2024 and in June 2025 in Castellammare di Stabia (40°43′47.3″ N, 14°28′46.9″ E; 3 m a.s.l.) in a suburban horticultural and floricultural area. Moths were also collected by sweeping spontaneous herbaceous vegetation with an entomological net inside an abandoned orchard located in Cercola (40°51′21.1″ N, 14°21′45.1″ E, 96 m a.s.l.), in August 2024 and in a suburban orchard in San Gennaro Vesuviano (40°52′04.0″ N, 14°31′19.9″ E; 56 m a.s.l.) in November 2024. Finally, moths were sampled by collecting young herbaceous plants (*Conyza* spp., of the Asteraceae family). These plants, after being inspected for the presence of larval damage on the leaves and transplanted into pots, were grown inside rearing cages under field conditions. Initially, *Conyza* plants were collected and caged for laboratory purposes other than those related to *P. stultana*. After observing that *P. stultana* adults emerged inside the cages, evidently originating from eggs and larvae present on wild vegetation, we collected *Conyza* plants specifically to monitor the presence of the moth. Sampling was carried out in several locations: Santa Anastasia (40°53′31.4″ N, 14°22′48.9″ E; 41 m a.s.l.), on the margins of an agro-forestry area, from November 2023 to April 2024 and from June to September 2025; Portici (40°49′39.6″ N, 14°20′42.8″ E; 80 m a.s.l.), in an urban garden, from May to October 2024; and San Giorgio a Cremano (40°50′03.1″ N, 14°21′07.2″ E; 85 m a.s.l.), in a suburban horticultural and fruit-growing area, from May to September 2025. The cages were checked twice a week, and the adult specimens were counted as soon as they emerged from the pupal stage.

### 2.2. Taxonomic Identification and Molecular Analysis

**Figure 2:**
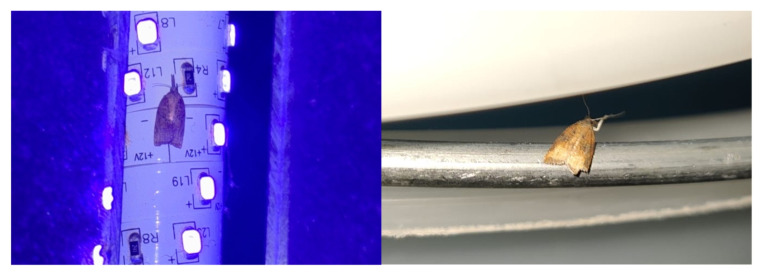
*Platynota stultana* specimens attracted to a UV light trap. One individual landed on the LED light (**left**), while the other landed on the bucket of the trap (**right**).

**Figure 3:**
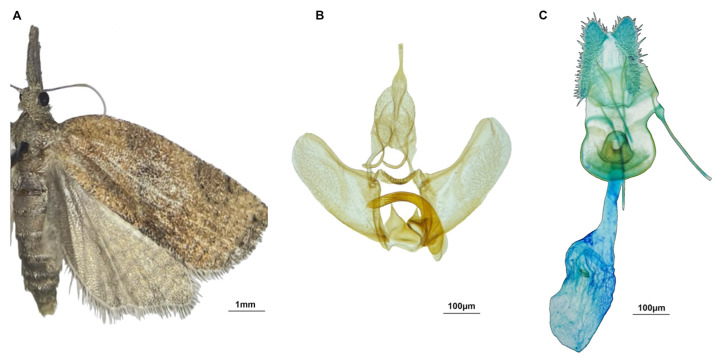
*Platynota stultana*: adult male specimen (**A**), and male (**B**) and female (**C**) genitalia.

Adult specimens were prepared according to the Zimmerman protocol [20] with slight modifications: they were softened in a humid chamber for 15–18 h, then pinned, positioned on spread boards, labelled, and, after drying, stored in entomological boxes. Adult specimens of *P. stultana* (Figure 2 and Figure 3) display a bell-shaped appearance with the wings held either flat or roof-like over the abdomen [21]. Moreover, they show long, grey labial palps, a trait uncommon within Tortricidae but present in species belonging to the tribe Sparganothini [8,22]. Male and female genitalia (Figure 3) were dissected and mounted on slides according to Robinson’s [23] protocol. Identification at the species level was carried out using the genital characteristics and by following relevant taxonomic keys [12,22,24].

The taxonomic identification of adult moths, based on morphological traits, was followed by molecular characterization. DNA was extracted from a single leg of each insect using a Chelex-proteinase K protocol, as reported by Gebiola et al. [25]. The barcoding region of the mitochondrial gene cytochrome oxidase c subunit I (COI) was sequenced. The COI gene was amplified using the standard primer pair LCO1490 and HCO2198 [26]. Amplification was carried out according to the protocol used by Goglia et al. [27]. Each reaction was performed in a 50 μL volume containing 1.25U Taq DNA Polymerase recombinant and 5 μL of 10X Taq buffer (Thermo Fisher Scientific, Waltham, MA, USA), 3 μL of 1.5 mM MgCl_2_, 2 μL of 5 mM dNTPs, 2.5 μL of 10 mM of each primer, and 1 μL of DNA. The thermal cycling parameters included an initial denaturation at 94 °C for 1 min, followed by 40 cycles of denaturation at 94 °C for 30 s, annealing at 48 °C for 120 s, and extension at 72 °C for 60 s. One cycle of a final extension for 7 min at 72 °C was performed. Amplicons were sequenced in both directions by using standard Sanger sequencing services (Macrogen Europe, Amsterdam, The Netherlands). The chromatograms obtained were viewed and edited in Chromas v.2.6.4 (Technelysium, South Brisbane, Queensland, Australia). COI sequences were verified for protein-coding frameshifts and nonsense codons using MEGA 12 [28]. Overall, 11 adults (7 males and 4 females), representative of five sampling locations, were sequenced. COI sequences were compared with known sequences deposited in the GenBank database using the BLAST tool (https://blast.ncbi.nlm.nih.gov/Blast.cgi, accessed on 12 January 2026) and in the BOLD database (https://v4.boldsystems.org/index.php, accessed on 12 January 2026). Phylogenetic analysis of COI nucleotide sequences was performed using a Maximum-Likelihood (ML) framework. COI sequences were aligned using MAFFT v7 [29], and the resulting alignment was manually inspected and trimmed to ensure there were sequences of equal length (577 bp) for subsequent analyses. Evolutionary model selection was conducted with ModelTest-NG under a configuration compatible with RAxML-NG v0.9.0 [30]. Based on the AIC and AICc criteria, the GTR + I + G4 model was selected as the best fit for describing the substitution pattern. All codon positions were included in the analysis. The ML tree was inferred with RAxML-NG v0.9.0 [30], exploring tree space from multiple starting topologies, including ten parsimony-based and ten randomly generated trees. Branch support was assessed using 1000 nonparametric bootstrap replicates. A fixed random seed was used in RAxML-NG to ensure the reproducibility of the phylogenetic results. Phylogenetic analysis was performed, including in the alignment the sequences of the three *P. stultana* haplotypes we found in Southern Italy (see the Results Section 3.2), the sequences of *P. stultana* from North America retrieved from the BOLD database, and the sequences of 17 other *Platynota* species, which were also retrieved from BOLD. No sequences of *P. stultana* specimens collected in Europe were found in the BOLD database. No sequences of *P. stultana* were available in GenBank. Trees were rooted using *Sparganothis pulcherrimana* (Walsingham), *Sparganothis lycopodiana* (Kearfott), *Amorbia emigratella* Busck, and *Amorbia humerosana* Clemens—belonging to the tribe Sparganothini, as with *Platynota*—as outgroup taxa [31,32]. Overall, 65 *P. stultana* COI gene sequences were found in the BOLD database, of which 15 were not included in our analysis because they were too short or of poor quality. The remaining 50 sequences, trimmed to 577 bp, included 11 haplotypes, 10 of which were reported in BOLD as originating from specific geographic areas (Florida, California, Arizona, or New Mexico). A representative sequence for each of the 10 haplotypes was included in the alignment. Only one haplotype was reported in BOLD as being from two different geographic areas, namely, Arizona and Florida, and a representative sequence from each of the two areas was included in the alignment. The BOLD identification number of each of the 12 selected sequences of *P. stultana* is reported in the phylogenetic tree (see the Results, Section 3.2). Additionally, a Neighbor-Joining (NJ) phylogenetic tree was generated in MEGA 12 [28] using the TN93 + G model to compute evolutionary distances and 1000 bootstrap replications to assess node support. *Sparganothis pulcherrimana* and *S. lycopodiana* were used as outgroups. Genetic distance (uncorrected p-distance, that is, the number of base differences per site) between sequences was calculated using MEGA 12 [28].

## 3. Results

### 3.1. Insects Collected

A total of 37 adult *P. stultana* specimens (31 males and 6 females) were collected from May to November 2024 in five locations in the region of Campania, Southern Italy (Table 1, Figure 1). Both males and females were collected in Santa Anastasia and Castellammare di Stabia, while one female was collected in Cercola and only males were found in Portici and San Gennaro Vesuviano. All three collection methods used allowed for the sampling of adults from both sexes. In 2025, *P. stultana* was recovered in two localities sampled the previous year and at a new site, San Giorgio a Cremano, only 1 km from the Portici collection site (Table 1, Figure 1). Growing naturally infested wild *Conyza* plants, collected in Santa Anastasia, and San Giorgio a Cremano, led to the emergence of hundreds of adults in rearing cages from May to September 2025. In Castellammare di Stabia, two adults were collected with UV light traps. The collected specimens have been deposited in the entomological collection of the CNR-Institute for Sustainable Plant Protection (CNR-IPSP) in Portici (NA). Following our findings, the presence of *P. stultana* was formally communicated by the Plant Protection Service of Campania Region to the European Commission in accordance with Article 29 of Legislative Decree No. 19 of 2 February 2021 (Europhyt notification 2917, dated 22 November 2024).

### 3.2. Molecular Analysis

**Figure 4:**
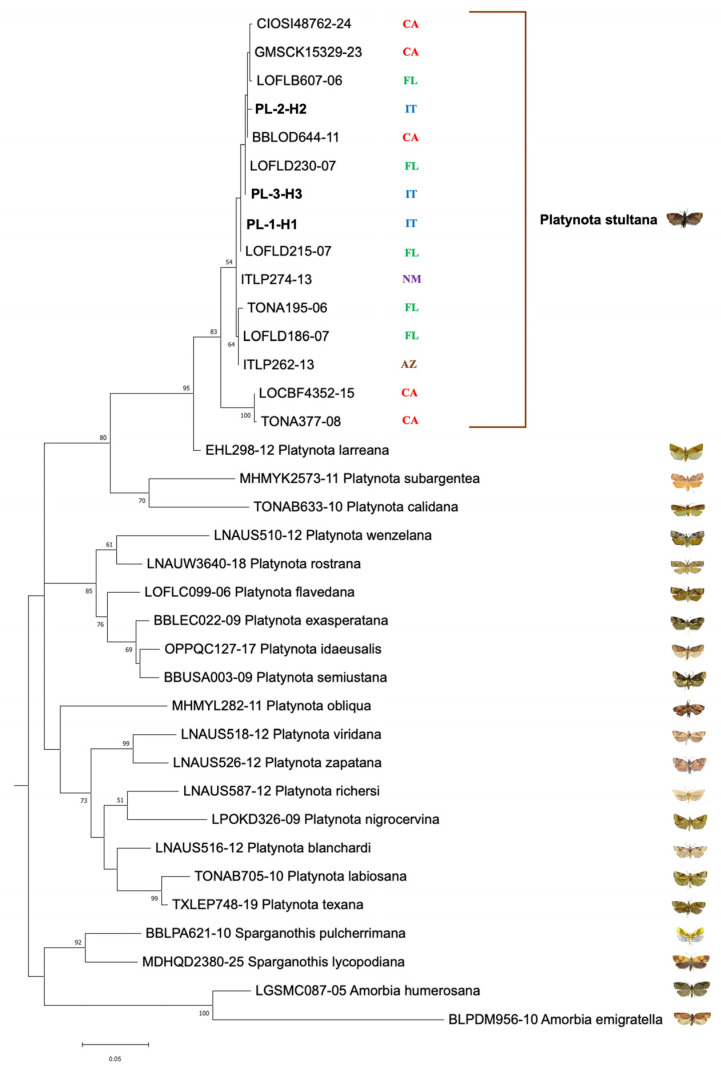
Maximum-likelihood phylogenetic tree for COI gene sequences of *Platynota* species. Italian haplotypes of *P. stultana* sequenced in this work are written in bold. Other sequences included in the analysis are identified by their identification number in the BOLD database. Geographic origins of *P. stultana* sequences are reported (AZ, Arizona; CA, California; FL, Florida; IT, Italy; and NM, New Mexico). Bootstrap values > 50% are shown on nodes. Photo credits: All images © [4,33,34,35,36,37,38], except the image of *P. stultana* © [Lorenzo Goglia].

Analysis of the COI gene sequences (577 bp) of 11 adult *P. stultana* specimens from Southern Italy revealed three haplotypes whose distribution was not specifically associated with the sampling location. Haplotype H1 was the most frequent (7/11 individuals), followed by H3 (3/11 individuals) and H2 (only 1 individual) (Figure 1, Table S1). Genetic distance (as uncorrected p-distance, that is, the number of base differences per site) varied between the three haplotypes from 0.35% to 0.87% (Table S2). The sequences have been deposited in the GenBank database under accession numbers PQ585102 to PQ585106 and PV235054 to PV235059. Compared with the *P. stultana* sequences deposited in the BOLD database, Italian haplotypes H1 and H3 show 100% identity with sequences from Florida. The H2 haplotype shows the highest degree of genetic identity (99.65%) with respect to a haplotype from California. Furthermore, all three haplotypes showed a high level of identity, ranging from 95.84% to 99.83%, with respect to other haplotypes from Florida, California, Arizona, and New Mexico. There were no *P. stultana* sequences from outside North America deposited in the database. A BLAST search in the GenBank database produced the closest match (96.20% identity) with a sequence of *Platynota larreana* (Comstock). ML phylogenetic analysis produced a tree (Figure 4) showing that *P. stultana* sequences are grouped into a highly supported clade, whose closest relative was *P. larreana*. Within the *P. stultana* clade, sequences are grouped into two supported clades. The main one includes sequences with relatively low genetic variation. Indeed, the uncorrected p-distance between sequences from Southern Italy, Florida, California, Arizona, and New Mexico ranged between 0.0% and 1.39% (Table S2). The other clade comprises two nearly identical sequences that diverge from those of the main clade by 3.12–4.16% (uncorrected p-distance) (Table S2). NJ analysis produced a tree (Figure S1) with a topology almost identical to the ML tree. The genetic distance between *Platynota* species ranged between 2.08% and 11.96% (uncorrected p-distance) (Table S2).

## 4. Discussion

Since the early twentieth century, the intensification of global trade and travel has facilitated the movement of numerous species across different regions of the world [39]. At the same time, climate change has altered environmental conditions, weakening ecological barriers and promoting the spread and establishment of exotic species in new habitats [40]. Invasive alien species impose substantial ecological and economic burdens worldwide. In Europe, overall losses for agriculture, forestry, and fisheries due to biological invasions have been estimated to have exceeded $140 billion between 1960 and 2020, and in the Mediterranean basin alone, they have been estimated to amount to $27.3 billion over the past three decades [41]. Europe hosts the highest concentration of alien organisms, with Italy and France emerging as key entry and distribution areas due to their geographical positions and extensive trade links with other European and global countries [42]. The American continent represents the place of origin of dozens of invasive pests that have been introduced into Europe over time, causing significant economic losses for the agricultural sector [43]. In this context, there is a high risk that a Nearctic pest such as *P. stultana*, which can travel with numerous agricultural commodities, due to its wide plant-host range [6], could be introduced into a new area. In the recent past, several species of Lepidoptera from the American continent have been introduced into Europe through trade routes. The most emblematic case is that of the Neotropical species *Tuta absoluta* (Meyrick), which, after being introduced into Spain from South America in 2006, spread within a few years to all Mediterranean countries, eventually becoming a major pest of tomato crops globally [44,45]. Countries in the Mediterranean basin are at risk of invasion by other Lepidoptera species, such as the Neotropical *Spodoptera frugiperda* (Smith), whose distribution is still limited in some European countries but is increasingly expanding in the Southern Hemisphere, posing a potential threat to the food chains of Africa, Asia, and Oceania [46]. *Platynota stultana* had joined the list of potentially invasive species. It has been reported repeatedly in several European countries since 2004. First intercepted in UK, this species has been officially recorded in France, Germany, Netherlands, Malta, Spain, and Italy [6]. However, to date, the only country where *P. stultana* appears to be established is Spain, following its introduction in (probably) 2005 [12,17]. In Italy, *P. stultana* was first reported as an occasional capture using sex pheromone traps in 2022 in Apulia [13,18]. Subsequently, the moth has been reported several times in the southern (Apulia and Sicily), central (Latium and Tuscany), and northern (Liguria) regions [16], but most of these records were isolated reports, mostly retrieved from citizen science databases (e.g., Lepiforum e. V., iNaturalisr.org). Consequently, it is unclear if these records represent transient findings or are the result of the species’ establishment.

In this study, we recorded the presence of *P. stultana* in the Campania region, Southern Italy, for the first time and contextually showed that the species is established in this region. We provided several lines of evidence to support this finding: adults of both sexes were sampled in different locations and under different environmental conditions; adults were recorded in two consecutive years and in increasing numbers; and adults emerged inside cages containing wild potted *Conyza* plants, suggesting that *P. stultana* is able to reproduce in the field and use *Conyza* plants as hosts. Our data, combined with previous observations [13,16], suggest that *P. stultana* is expanding its invasion range into Southern Italy, where the pest may find a warm climate, similar to that of its native range [47], and wide availability of host plants

Sequencing the mitochondrial COI gene of *P. stultana* allowed us to identify three haplotypes distributed among the populations sampled in the Campania region, suggesting the alien pest may have been introduced multiple times. The H1 haplotype—the most frequent one (64%), having been found in four out of five collection sites—could represent the first introduction in the studied area. The H3 haplotype, with a frequency of 27%, was found in three out of five sites and always in combination with H1. Furthermore, both the H1 and H3 haplotypes have 100% genetic identity with respect to haplotypes from Florida. Consequently, it is possible that the simultaneous introduction of H1 and H3 also occurred. The single individual with the H2 haplotype, which shows the highest genetic identity (99.65%) with a haplotype from California, suggests that multiple introductions may have occurred over time. Overall, our data suggest that the population of *P. stultana* sampled in Campania predominantly originates from Florida as a result of a direct introduction or redistribution from other countries, e.g., Spain, where *P. stultana* is mainly associated with crops of pepper [12], a vegetable widely exported to Italy.

Phylogenetic analysis of the COI barcoding region showed that the *P. stultana* clade, whose closest relative was *P. larreana*, splits into two subclades. The main one includes sequences, with relatively low genetic variation, from Southern Italy, Florida, California, Arizona, and New Mexico. The other subclade comprises two nearly identical sequences from California, which diverge from those of the other subclade by 3.12–4.16% (uncorrected p-distance). Given that a divergence threshold of 2–3% for the COI gene is commonly used as a preliminary indicator of potential species-level differentiation [48,49,50], and that interspecific distances between *Platynota* species range from 2.08% to 11.96%, it is possible that the *P. stultana* clade may also include a cryptic species. Its taxonomic identity will need to be clarified through further genetic data and an in-depth morphological analysis.

Regarding the methods used in this study to collect *P. stultana*, sampling wild *Conyza* plants and subsequently rearing them in cages proved to be very effective, allowing for the capture of large numbers of adults. *Platynota stultana* is known to use several species of Asteraceae as host plants [5,6], and *Conyza* is an example. At least in the studied area of Southern Italy, *Conyza* plants seem to be a preferred host for oviposition and larval feeding, and their sampling could be an effective method of assessing the distribution of the pest in combination with UV light traps [51] or sex pheromone traps [47,52]. In our study, UV light traps also performed well, allowing us to capture a considerable number of adults, mainly males (90%), as already reported [51].

Currently, *P. stultana* probably does not pose an immediate threat in the European countries where it is established. In Spain, no significant damage has been reported in pepper crops since the insect was first detected in 2009. In Southern Italy, to date, no reports of its presence in crops have been reported. However, considering the climate projection for the Mediterranean Region, it is expected that this area will be severely affected by global warming, with an increase in average annual temperatures of 1.5–2.5 °C by 2041–2070 [53]. In this scenario, a thermophilic species such as *P. stultana* could find optimal conditions over time, conditions increasingly similar to those of its native range, which could improve its survival during the winter and increase the number of annual generations. Consequently, its invasion range will likely continue to expand in Italy and other Mediterranean countries, where *P. stultana* could become an economic pest. Effective monitoring over time will be necessary to quantify potential crop damage and implement timely control measures for *P. stultana* in order to avoid disastrous economic impacts such as those experienced in the recent past following the invasion of *T. absoluta*.

## Figures and Tables

**Table 1:** Sampling of *Platynota stultana* in 2024–2025 in the Campania region, Southern Italy.

Location	Coordinates N, E	Collection Date	Collection Method	N° Adults Sampled	
				Females	Males
Cercola	40.855861, 14.362528	28 August 2024	Sweeping	1	0
Santa Anastasia	40.892056, 14.380250	9 May 2024	Wild *Conyza* plants in cage	0	1
Santa Anastasia	40.892056, 14.380250	5 July 2024	Wild *Conyza* plants in cage	0	1
Santa Anastasia	40.892056, 14.380250	26 August 2024	Wild *Conyza* plants in cage	2	0
Santa Anastasia	40.892056, 14.380250	May–September 2025	Wild *Conyza* plants in cage	25	31
Portici	40.827667, 14.345222	16 October 2024	Wild *Conyza* plants in cage	0	1
Portici	40.827667, 14.345222	7 November 2024	Wild *Conyza* plants in cage	0	1
San Giorgio a Cremano	40.834194, 14.352000	May–September 2025	Wild *Conyza* plants in cage	48	40
Castellammare di Stabia	40.729806, 14.479694	16 October 2024	UV Light Trap	3	11
Castellammare di Stabia	40.729806, 14.479694	24 October 2024	UV Light Trap	0	15
Castellammare di Stabia	40.729806, 14.479694	4 June 2025	UV Light Trap	0	2
San Gennaro Vesuviano	40.867764, 14.522183	25 November 2024	Sweeping	0	1

## Data Availability

The original contributions presented in this study are included in the article/Supplementary Material. Further inquiries can be directed to the corresponding authors.

## Supplementary Materials

The following supporting information can be downloaded at https://www.mdpi.com/article/10.3390/insects17010122/s1, Supplementary Table S1: COI gene haplotypes of *Platynota stultana*, sequenced from individuals collected in the Campania region, Southern Italy. Supplementary Table S2: Uncorrected *p*-distance (number of base differences per site) between COI gene sequences of *Platynota* species. Supplementary Figure S1: Neighbor-Joining phylogenetic tree for COI gene sequences of *Platynota* species. Reference [54] is cited in the Supplementary Materials.
