# Supplementary material for: The Invasive Nearctic Pest Platynota stultana Walsingham (Lepidoptera: Tortricidae) Is Established in Southern Italy
Source: Insects. 2026 Jan 21;17(1):122. doi: 10.3390/insects17010122 (PMC12842250; doi:10.3390/insects17010122)
Supplement: Supplementary file 1 [file insects-17-00122-s001.zip › Figure S1.pdf]

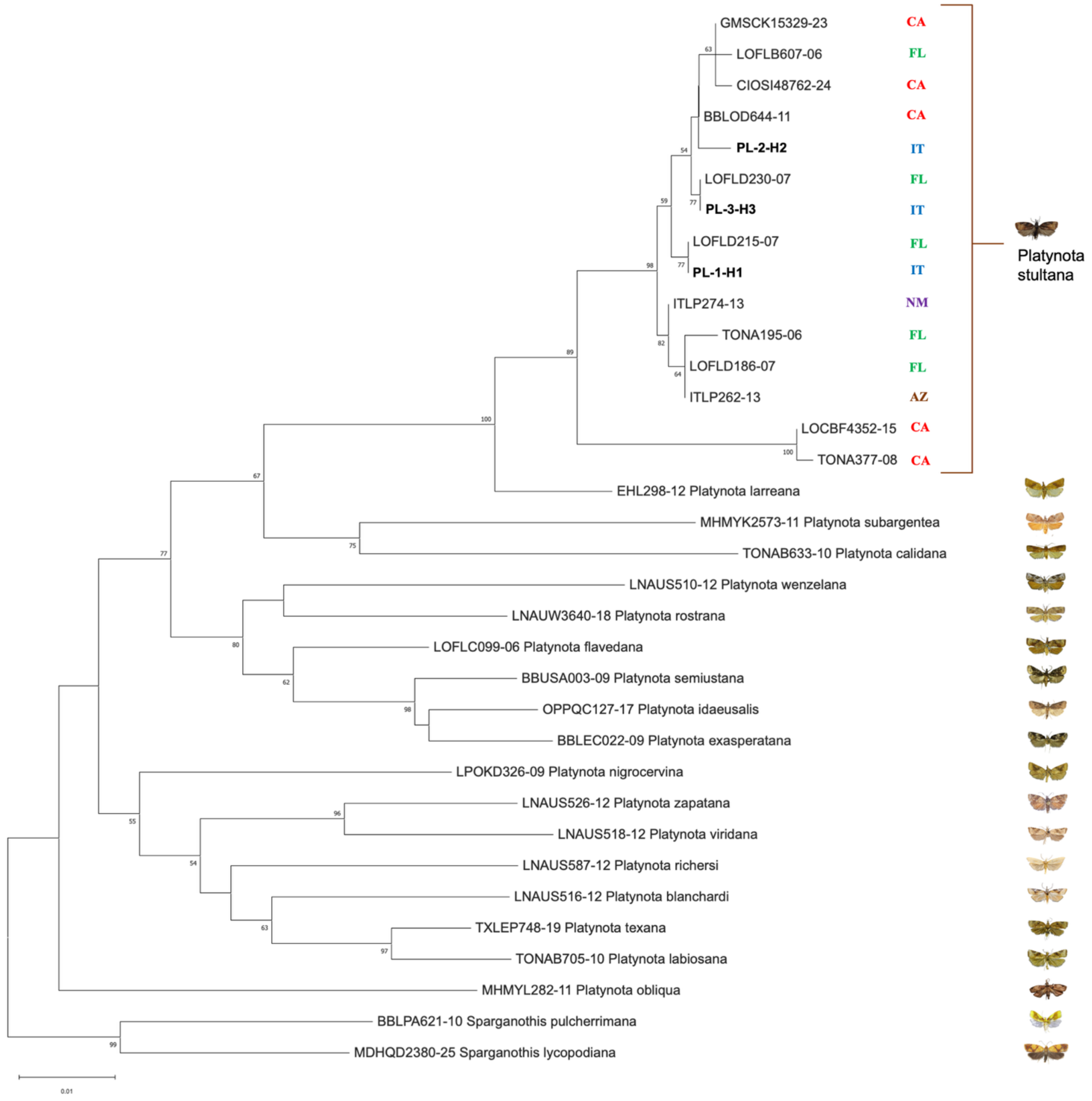

Figure S1. Neighbor-Joining phylogenetic tree for COI gene sequences of *Platynota* species. Italian haplotypes of *P. stultana* sequenced in this work are reported in NCBI. Other sequences included in the analysis are identified by their identification number in the BOLD database. Geographic origin of *P. stultana* sequences is reported (AZ, Arizona; CA, California; FL, Florida; IT, Italy; NM, New Mexico). Bootstrap values >50% are shown on nodes. Photo credits: All images © [4, 33, 34, 37, 38], except image of *P. stultana* © [Lorenzo Goglia].
