# Supplementary material for: The Invasive Nearctic Pest Platynota stultana Walsingham (Lepidoptera: Tortricidae) Is Established in Southern Italy
Source: Insects. 2026 Jan 21;17(1):122. doi: 10.3390/insects17010122 (PMC12842250; doi:10.3390/insects17010122)
Supplement: Supplementary file 1 [file insects-17-00122-s001.zip › Table S1.pdf]

Table S1. COI gene haplotypes of *Platynota stultana*, sequenced from individuals collected in the Campania region, southern Italy

ID Sample	Site	Coordinates N, E	Collection date	Collection method	Sex	COI haplotype	GenBank Accession Number
PL 1	Santa Anastasia	40.892056 14.380250	9.V.2024	Wild plants in rearing cage	Male	H1	PQ585102
PL 2	Cercola	40.855861 14.362528	28.VIII.2024	Sweeping	Female	H2	PQ585103
PL 3	Santa Anastasia	40.892056 14.380250	28.VIII.2024	Wild plants in rearing cage	Female	H3	PQ585104
PL 4	Santa Anastasia	40.892056 14.380250	26.VIII.2024	Wild plants in rearing cage	Female	H1	PQ585105
PL 5	Santa Anastasia	40.892056 14.380250	5.VII.2024	Wild plants in rearing cage	Male	H1	PQ585106
PL 16	Portici	40.827667, 14.345222	16.X.2024	Wild plants in rearing cage	Male	H1	PV235054
PL 17	Castellammare di Stabia	40.729806 14.479694	16.X.2024	UV Light Trap	Female	H3	PV235055
PL 21	Castellammare di Stabia	40.729806 14.479694	15.X.2024	UV Light Trap	Male	H1	PV235056
PL 30	Castellammare di Stabia	40.729806 14.479694	24.X.2024	UV Light Trap	Male	H1	PV235057
PL 35	Portici	40.827667, 14.345222	7.XI.2024	Wild plants in rearing cage	Male	H3	PV235058
PL 37	San Gennaro Vesuviano	40.867764 14.522183	25.XI.2024	Sweeping	Male	H1	PV235059
